# An Innovative Smart and Sustainable Low-Cost Irrigation System for Anomaly Detection Using Deep Learning

**DOI:** 10.3390/s24041162

**Published:** 2024-02-10

**Authors:** Rabaie Benameur, Amine Dahane, Bouabdellah Kechar, Abou El Hassan Benyamina

**Affiliations:** 1 Research Laboratory in Industrial Computing and Networks (RIIR), University of Oran 1, B.P. 1524, El M’Naouer, Oran 31000, Algeria; benameur.rabaie@edu.univ-oran1.dz (R.B.); kechar.bouabdellah@univ-oran1.dz (B.K.); 2 Institute of Applied Science and Technology, ISTA, University of Oran 1, B.P. 1524, El M’Naouer, Oran 31000, Algeria; 3 Laboratory of Parallel, Embedded Architectures and Intensive Computing (LAPECI), University of Oran 1, B.P. 1524, El M’Naouer, Oran 31000, Algeria; benyamina.hassen@univ-oran1.dz

**Keywords:** IoT, smart farming, anomaly detection, low cost, fog-AI, deep learning

## Abstract

The agricultural sector faces several difficulties today in ensuring the safety of food supply, including water scarcity. This study presents the design and development of a low-cost and full-featured fog-IoT/AI system targeted towards smallholder farmer communities (SFCs). However, the smallholder community is hesitant to adopt technology-based solutions. There are many overwhelming reasons for this, but the high cost, implementation complexity, and malfunctioning sensors cause inappropriate decisions. The PRIMA INTEL-IRRIS project aims to make digital and innovative agricultural technologies more appealing and available to these communities by advancing the intelligent irrigation “in-the-box” concept. Considered a vital resource, collected data are used to detect anomalies or abnormal behavior, providing information about an occurrence or a node failure. To prevent agro-field data leakage, this paper presents an innovative, smart, and sustainable low-cost irrigation system that employs artificial intelligence (AI) techniques to analyze anomalies and problems in water usage. The sensor anomaly can be detected using an autoencoder (AE) and a generative adversarial network (GAN). We will feed the autoencoders’ anomaly detection models with time series records from the datasets and replace detected anomalies with the reconstructed outputs. When integrated with an IoT platform, this methodology is a tool for easing the labeling of sensor anomalies and can help create supervised datasets for future research. In addition, anomalies can be corrected by prediction models based on deep learning approaches, applying CNN/BiLSTM architecture. The results show that AEs outperform the GANs, achieving an accuracy of 90%, 95%, and 97% for soil moisture, air temperature, and air humidity, respectively. The proposed system is designed to ensure that the data are of high quality and reliable enough to make sound decisions compared to the existing platforms.

## 1. Introduction

**Figure 1:**
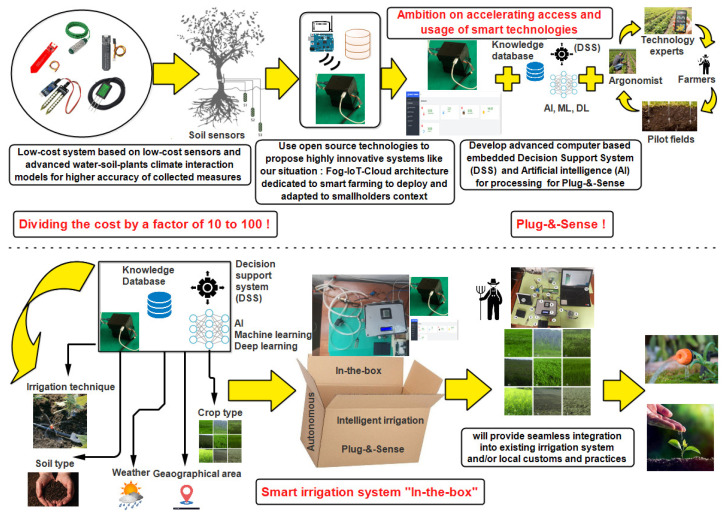
“Intelligent irrigation in a box” idea of PRIMA INTEL-IRRIS project.

The world’s population is anticipated to increase by close to 2 billion by 2050, causing a rapid escalation in food demand. To cater to the needs of the increasing population, the agricultural industry needs to be modernized, smart, and automated [1]. The food and agriculture organization (FAO) [2] claims that small-scale farming significantly contributes to rural economies and food security. On the other hand, smallholders usually deal with a range of restrictions that reduce their ability to produce profitably and advance the economy [3]. However, smallholder farmer communities (SFCs) have not benefited from adopting solutions to increase irrigation efficiency because of the high starting costs and the high skill levels needed to understand the technology. Recently, the fourth industrial revolution (IR 4.0) paradigm shift has ushered in a surge in technological innovation and sustainable transformation across various industries and sectors [4]. The innovative use of printed sensors for measurement and monitoring in smart agricultural applications has garnered considerable attention. Smart farming utilizing printed sensors is in its infancy and requires additional research [5]. Significant limitations restrict SFCs’ productivity and profit potential. As a result of a variety of crucial issues, including a lack of agricultural expertise, financial resources, climate change, and market access to address these challenges, we propose in this research a low-cost (USD 260) smart farming system for monitoring and prediction that will assist SFCs in forecasting future data in order to compute their water demands and properly schedule irrigation. The sensor component employs a low-cost design that is basic but sturdy and efficient, inspired by various do-it-yourself (DIY) initiatives and previous contributions [6,7]. In terms of accuracy and error reduction, the system outperforms others. In this context, the goal of the PRIMA INTEL-IRRIS project [8] is to increase the visibility and accessibility of digital and smart agricultural technologies among small and medium-sized farms. To meet the needs of this community, the suggested solutions must be economical, easy to implement in the field, and, most importantly, adaptable to current agricultural techniques. As a result, by inventing a low-cost, smart irrigation control system, INTEL-IRRIS hopes to transform the view that SFCs have of what was formerly highly expensive technology. INTEL-IRRIS also hopes that by using the “intelligent irrigation in a box” idea, they can make smart irrigation systems as easy to set up and use as home appliances, with a small investment compared to how much money they make. The main objective of this research is to improve on our previous work [3,9] by increasing the accuracy of low-cost sensors for data collection so that our water–soil–plants–climate interaction models will provide increased accuracy of predicted environmental factors to enhance the irrigation efficiency of smallholder farmers. There is a current need for our low-cost fog-IoT/AI system, version 1.0, that employs deep learning to analyze in real-time the anomalies and problems that appear in low-cost sensors. To reach this goal, we propose a low-cost, sustainable irrigation system that helps smallholder farmers manage irrigation more efficiently by providing a low-cost, open-source, autonomous, and easy-to-use smart irrigation control system that takes advantage of IoT capabilities, as shown in Figure 1.

We will implement the “Intelligent Irrigation in a Box” idea by presenting a sensor/ control/actuator-based “plug-and-sense” system using significant technological advances from the last few years, including IoT, artificial intelligence (AI), and decision support systems (DSS). This technology may be linked with existing irrigation infrastructure [10,11], allowing it to be controlled according to smallholders’ customs. Using sensor technologies, the provided platform collects critical physical phenomena such as soil moisture, air temperature, air humidity, water level, water velocity, and light intensity using a DL methodology. An unsupervised learning approach is well-suited for detecting anomalies, especially in cases with limited labeled time-series samples and complex, nonlinear climate and soil patterns. According to the literature, the most promising approaches for anomaly detection are Autoencoders [12] and GANs [13] in time series. The sliding window technique is highly appropriate for predicting future sensor readings through a supervised learning approach. Utilizing a hybrid deep learning architecture, combining convolutional and recurrent neural networks, enables the attainment of a minimal estimation error for similar sequence-to-sequence prediction problems [14]. Consideration is given to a novel Fog-IoT-Cloud platform for storing, analyzing, and using vast volumes of acquired data to assist SFCs in managing the irrigation mechanism efficiently.

This work’s main contributions are as follows:

(i) The design and development of a low-cost fog-IoT/AI system, version 1.0, fully targeted towards smallholder farmer communities (SFCs) to enhance irrigation decision making.
(ii) We provide a comprehensive performance assessment and comparison between the autoencoders (AE) and generative adversarial networks (GAN) to detect anomalies in environmental factors data.
(iii) Predicting the most important environmental factors (air temperature, air humidity, soil moisture) based on field sensory data and weather forecast data using CNN/BiLSTM architecture to provide SFCs with recommendations.

The rest of the paper is structured as follows: Section 2 presents a review of the relevant literature on irrigation systems. The INTEL-IRRIS fog-IoT/AI system, at the heart of the architectural design discussed in this paper, will be presented in Section 3. Sensor anomaly detection for smart irrigation systems is provided in Section 4. Section 5 describes the most important environmental factors that can be predicted using deep learning. Finally, conclusions and future trends are given in Section 6.

## 2. Related Studies

In this section, we present a review of the relevant literature on irrigation systems. Each of them has focused on various aspects of the irrigation task. As illustrated in Table 1, we categorize the studies based on the following criteria:

(i) Underground sensing: the IOUT requirement emerges partially or fully buried underground for real-time monitoring and soil sensing; several essential irrigation characteristics include soil moisture, soil texture, soil salinity, etc.
(ii) Environmental sensing: it is used to monitor important weather pattern changes in real-time.
(iii) Geographic information systems (GIS): provide a potent instrument for near-real-time analysis of crop status. Ground-based equipment, aerial drones, and satellites all collect GIS agriculture data.
(iv) Soil texture: on precision agriculture (PA), we computed the useable reserve of water (RU) based on the soil texture (clay, limon, sand, organic matter).
(v) Machine learning approaches: it indicates whether the study considered machine learning approaches for intrusion detection systems.
(vi) Deep learning approaches: it specifies if the study was focused on deep learning approaches for intrusion detection systems.
(vii) Low-cost: the objective of the Intel-Irris project is to use low-cost sensors by dividing the cost by a factor of 10 to 100.
(viii) Anomaly detection: the detection of anomalies is a crucial stage that will enhance the quality of results in predictive modeling and pattern extraction, enabling the identification of problems and facilitating decision making in data-based services.

In our previous works [3,9], we suggested an Edge-IoT-Cloud platform based on a deep learning methodology for monitoring and predicting farmers’ ability to satisfy crop water demands when there is insufficient rainfall. Recently, Citoni et al. [15] published a review and analysis of currently available LoRaWAN-enabled Internet of Things (IoT) applications for intelligent agriculture. Vuran et al. [16] focus only on the underground factors; however, environmental sensing and SIG play a very important role in irrigation tasks. Optimizing water resources for agricultural production using an automated irrigation system has proven feasible and cost-effective, as proposed by Gutierrez et al. [17].

**Table 1:** A comparative analysis between the proposed platform and previous platform.

Ref	Underground Sensing	Environmental Sensing	GIS	Soil Texture	ML	DL	Low Cost	Anomaly Detection
[3]	Yes	No	Yes	Yes	Yes	Yes	Yes	Yes
[15]	No	Yes	No	No	No	No	No	No
[18]	No	Yes	No	No	Yes	Yes	No	No
[19]	Yes	Yes	No	No	Yes	No	No	No
[20]	Yes	No	No	Yes	Yes	No	Yes	No
[21]	Yes	Yes	No	Yes	No	No	Yes	No
[22]	No	No	Yes	No	Yes	No	No	No
[17]	Yes	Yes	No	No	No	No	No	No
[23]	No	Yes	No	No	Yes	No	No	No
[24]	Yes	No	No	No	No	No	Yes	No
Proposed platform	Yes	Yes	No	Yes	Yes	Yes	Yes	Yes

A few scientific studies focus on the application of data processing techniques to agricultural data in order to develop a more robust decision support framework. Roopaei et al. [22] presented an appropriate approach to identifying key parameters to schedule irrigation: the amount of water used was monitored, irrigation was scheduled on the basis of the canopy temperature distribution of the plant, and data were collected by means of thermal imaging. However, using the thermal imaging technique has many drawbacks that affect irrigation scheduling: different emissivities and reflections from surfaces obstruct precise temperature measurements, and most thermal imaging cameras have ±2% accuracy. Also, thermal images are difficult to interpret for specific objects with erratic temperatures. Aurora et al. [25], using unsupervised algorithms, devised and tested a three-step irrigation system for urban parks. By extracting prior knowledge using a multivariate approach, this methodology reduces the time required by univariate anomaly detection systems to analyze the entire univariate time series. Goap et al. [19] proposed a smart irrigation architecture based on IoT and a hybrid approach that relies on DL to predict soil moisture. The proposed algorithm uses sensor data collected from recent and past weather forecasts to predict soil moisture. However, environmental sensing is not sufficient to make a good irrigation schedule, and the authors also do not take into consideration the crop coefficient and the soil texture. Chen et al. [26] suggest a new approach for making irrigation decisions in paddy rice farming that uses deep Q-learning and short-term weather predictions. The new method was compared to the widespread practice of flooded irrigation in southern China. The results indicated the reliability of the daily rainfall forecast and the efficiency of the Deep Q-Learning strategy for saving water. Boursianis et al. [23] discussed the AREThOU5A IoT platform subsystems and their primary operations, as well as the deployed layered architecture stack. Within the context of the AREThOU5A IoT platform, an innovative method for delivering power to the platform’s IoT modules has been implemented. Cheema et al. [24] constructed an intelligent system with multiple sensors and devices related to internet of things (IoT) technologies. In addition, they developed an Android application called "Kistan Pakistan" that allows illiterate and low-literate farmers to remotely administer its functions. Pham et al. [20] described a low-cost and full edge-IoT/AI system targeting smallholder farmer communities and how it can provide the intelligent irrigation “in-the-box” concept. It offers an “out-of-the-box” function, meaning that the control component that recommends irrigation is integrated into the IoT gateway and, as a result, does not need an internet connection, as implemented by WAZIUP partner [27]. In contrast to previous research, the current study incorporates four factors to improve irrigation efficiency, productivity, quality, profitability, and the sustainability of agricultural production. In particular, we take into account the following factors: (i) soil texture; (ii) crop coefficient; (iii) sensed data with the weather forecast; and (iv) sensing of the subsurface parameter, in this instance, soil moisture. The objective is to calculate the requisite water quantity based on these variables.

## 3. Supplying Smallholder Farmers with Smart Technology

### 3.1. A Summary of the INTEL-IRRIS and PNR Projects

As stated earlier, INTEL-IRRIS [8] aims to make digital and intelligent agricultural technology more appealing and accessible to small and medium-sized farms. However, a compromise will be made to accomplish the primary aim: to create a low-cost irrigation system that SFCs can implement out of the box. The soil moisture sensor component follows a basic, resilient, and cost-effective design that has been substantially influenced by several do-it-yourself projects and prior contributions [6,20]. Unlike low-cost sensors that often offer unreliable data, INTEL-IRRIS will significantly improve the quality of collected data with (i) improved calibration of various sensors; (ii) the calculation of the needed amount of water based on the farm’s soil texture and crop coefficient; and (iii) the prediction of environmental factors based on field sensory data using DL.

### 3.2. Smart and Sustainable Irrigation System

**Figure 2:**
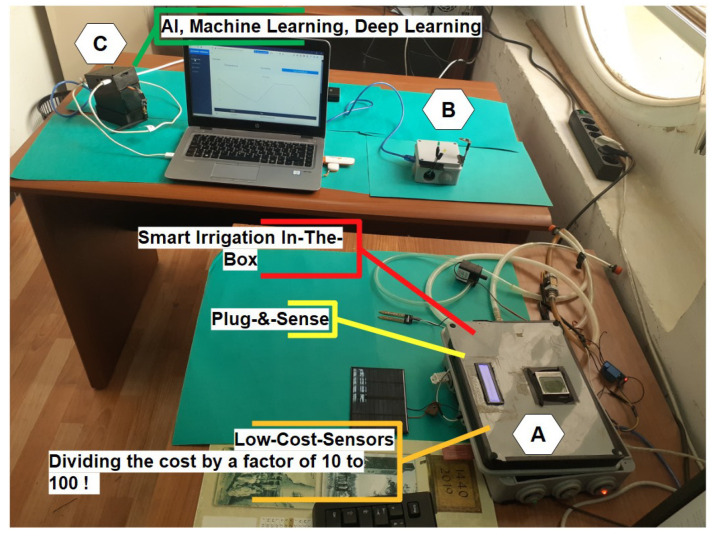
Global view of our proposed operational architecture.

Figure 2 shows how the smart farming system architecture collects, transfers, and processes physical parameters from small-scale farming, such as soil moisture, air temperature, air humidity, water level, water flows, the intensity of light, combustible gas, etc., in order to improve irrigation efficiency by deploying a low-cost, open, autonomous irrigation control system based on IoT and AI techniques [28]. The irrigation scheduling algorithm determines the amount of water required to maintain the maximum production potential without wasting water (i) for a particular crop, (ii) at a specified time, and (iii) for a specific soil type. The following section summarizes the proposed platform’s details.

### 3.3. Hardware Architecture

As illustrated in Figure 2, the network component of our platform is supplied via multi-hop communication between box A (the sensor layer) and box B (the gateway node) up to box C (the fog layer). Finally, a virtual machine (VM) is created on the Azure cloud to deploy multiple anomaly detection and prediction models. In addition to using NRF24L01 modules for wireless communication and Arduino boards as microcontrollers, we also intended to use Raspberry Pi 3 B+ boards as a fog layer.

### 3.4. Software Architecture

**Figure 3:**
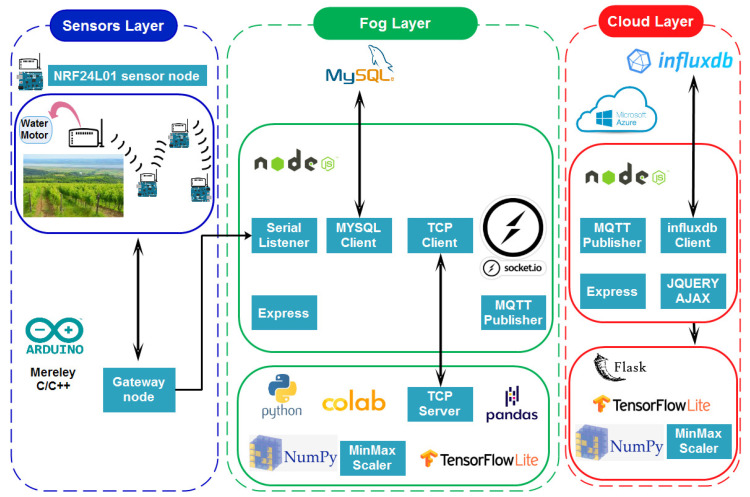
Global view of our software architectural proposal.

Using open-source development tools, we created a software architecture to reduce bandwidth, latency, cost, load balancing, and scalability, as shown in Figure 3. Using the Message Queuing Telemetry Transport (MQTT) protocol, communication between the gateway and server is established. The TCP transport layer protocol ensures dependable data transmission (COAP uses UDP). Because of this, it is encrypted with SSL, supports quality of service (QoS parameter 2: requires messages to be delivered exactly once), and has a much shorter message header than HTTP.

### 3.5. Box A: Sensing Node

The sensing node, which depicts the field data gathering tool, falls short of the total of USD 140. An Atmega328 microprocessor reads the output from these sensors. For around two dollars (USD), it may be used to construct a cheap design strategy for the Arduino.

**Figure 4:**
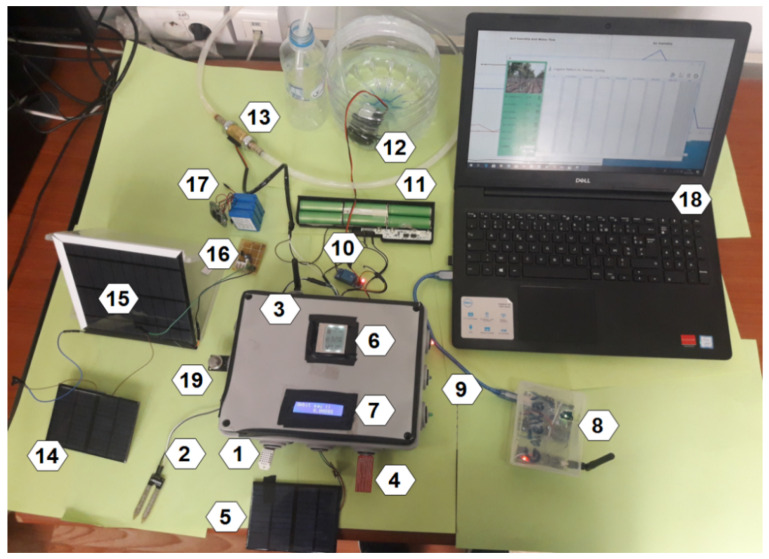
Global view of box A. Legends 1: DHT22 Sensor (USD 8); 2: soil moisture sensor (USD 5); 3: NRF24L01 module with adapter (USD 9); 4: water level sensor; 5: light sensor (USD 2); 6: LCD display nokia (USD 8); 7: LCD display I2C (USD 9); 8: relay node (USD 32), 9: LEDs notification (USD 2); 10: relay switch (USD 5); 11: power supply 12 V (USD 12); 12: water pump (USD 10); 13: water flow sensor (USD 9); 14: solar panel ZW85X115-12 (USD 2); 15: solar panel 6 V (USD 2); 16: amplifier (USD 5); 17: power supply 9 V (USD 10); 18: (PC) the mega Arduino card (USD 38) and NRF module are in the box.

As seen in Figure 4, this box’s sensor layer devices are designed to collect data from smallholder farmers and transmit it using the NRF24L01 radio module, which is available for roughly 5 dollars (USD) and enables the supply of a radio communication layer with an SPI interface. The farmer can examine the data and keep an eye on the crops in real-time since the data may be kept in a private database or in the cloud to construct a dataset.

Figure 4 provides a comprehensive description of the box’s sensors. We then set the NRF24L01 module to write mode and supply the destination address; we read the sensor values using the analogRead function on the data pins. The measured data are structured and transmitted to the IoT gateway every hour, where historical data are stored to detect anomalies at the fog layer. For the power supply of the sensor node, we used rechargeable 9 V batteries. Table 2 lists the cost of all components. In total, a sensing and actuation node costs USD 260. A large portion of the budget is for the cost of box C.

### 3.6. Box B: Relay Node

The relay node acts as an intermediate device and transmits the packets to the IoT gateway. In listening mode, we set up the gateway node by supplying the node address from whence the packets come, the channel number (122), and the data structure; if the data are received, it changes to sending mode, at which stage we provide the address of the next node. The power supply of the relay node was improved by using two AA batteries.

### 3.7. Box C: IoT Gateway

Fog computing facilitates the operation of computation, storage, and networking services between end devices and cloud computing. Box C permits data to be transmitted to the internet network, but not for a total of USD 90. We picked a Raspberry Pi 3 B+ type board since it has adequate hardware resources for processing and is based on the Linux kernel, making it compatible with most languages and AI libraries. It needs a 5 V power supply, an external Wi-Fi 802.11 card, and an Ethernet connector to connect to the internet. We used Python to create a concurrent TCP server (one thread per request), and we started by loading the prediction models from disk into the main memory (the kind of “*.tflite” files after the conversion to Tensorflow Lite). When a request comes in, a thread handles the receiving and preprocessing, resulting in the prediction. If we are looking for anomalies, we must compare the model outputs to an error threshold and provide the answer. On demand, we can also utilize the service to adjust the error levels of the various models. The server regularly delivers messages that include information such as the node’s IP address, available resources (RAM and CPU), and network latency. NodeJS listens on the serial port “/dev/tty0” at 115,200 baud. We extracted the measurements whenever new data were written to the port (separated by commas). We verified that all of the sensors were operational. This information is saved in the MySQL database for the time being. Finally, we produced a structured package including the latest six readings of each sensor, which had to correlate to the detection model anomaly entries. Then, we transmitted the latter to the Python-implemented service, which includes the various models; after receiving the answer, we checked for anomalies. An HTTP server configured using the “Express” module and listening on port 7000 sends the application pages to the browser. The route to each page is specified, and communication occurs via the “WebSocket” protocol. Every hour, a temporal event is triggered, which transmits the last received packet if none has come, allowing us to forecast it. The integrated Wi-Fi card is employed as an access point for configuration and adjusting the thresholds of anomaly detection models (leaving a means to reach the local network). Finally, data visualization from the gateway is required to assist the developer during maintenance. We investigated adding slave nodes that handle resource-intensive processing to enhance computing power and load balancing. The Raspberry Pi 3 B+ card’s Wi-Fi card creates a wireless network that the cluster uses to communicate.

### 3.8. Cloud Layer

**Figure 5:**
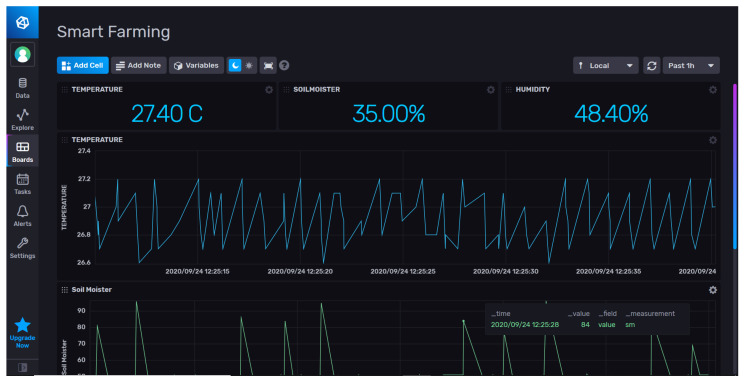
Monitoring and analysis of data on the cloud service.

We suggest using cloud services to store significant volumes of data generated by sensors in order to guarantee the accessibility and availability of our platform everywhere and at any time. Furthermore, we use them to anticipate future meters, ensuring scalability and more resources when our platform is overloaded (elasticity). A free student offer was provided to us, and we will utilize it to create a VM with a public IP address that we can access via the remote desktop protocol (RDP). As seen in Figure 5, Influx DB offers a limited free cloud service, so setting up a user’s dashboard for data analysis and monitoring is straightforward.

## 4. Sensor Anomalies Detection for Smart Irrigation Systems

Deep learning is becoming increasingly popular for tasks beyond image recognition, segmentation, and classification. Non-image data have been gaining more and more attention in recent years due to the advancement of IoT [29]. The main idea of unsupervised anomaly detection in time-series data is to identify whether data observations conform to normal data distributions over time. With this technique, we can discover patterns and anomalies that are otherwise hard to find. In order to spot unexpected patterns or anomalies as the data changes over time, it is crucial to comprehend how each variable within the time series affects the others.

**Figure 6:**
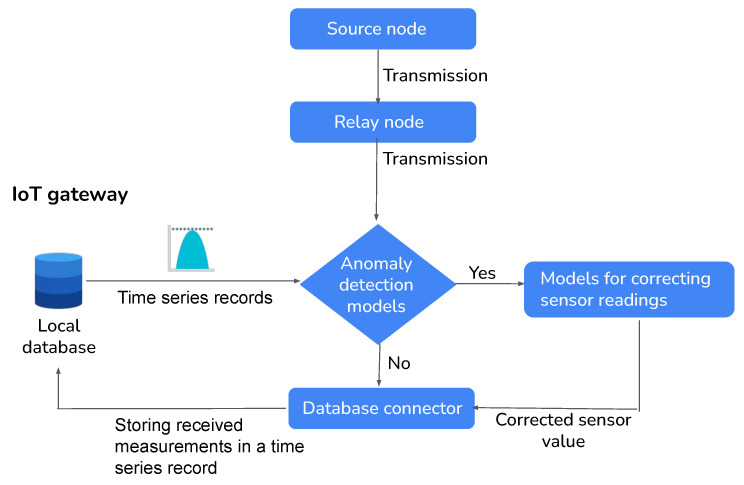
Illustration of IoT gateway sensor anomaly detection and correction process.

As shown in Figure 6, the received measurements are consolidated with the time series records in the local database. These merged data are used as input for anomaly detection models, which aim to identify any anomalies or unusual patterns within the data. The reconstructed inputs from the model are employed to estimate a corrected value for the abnormal measurement. The goal is to correct any inaccuracies or abnormalities identified in the original measurement. Once the new measure has been adjusted using the sensor value estimation model, it is stored back in the local database. This ensures that the corrected measure is available for future reference or analysis. In our research, the scarcity of labeled anomaly data poses a challenge. Therefore, GANs and AEs present advantageous options as they can effectively perform anomaly detection. Additionally, these techniques are capable of learning representations that capture both global and local dependencies within the data, enabling them to understand complex patterns effectively. GANs, in particular, leverage a discriminator network that can accurately classify anomalies. AEs focus on reconstructing input data and strive to minimize the reconstruction error. A threshold on the reconstruction error allows for the efficient identification of anomalies.

### 4.1. Generative Adversarial Networks (GAN)

**Figure 7:**
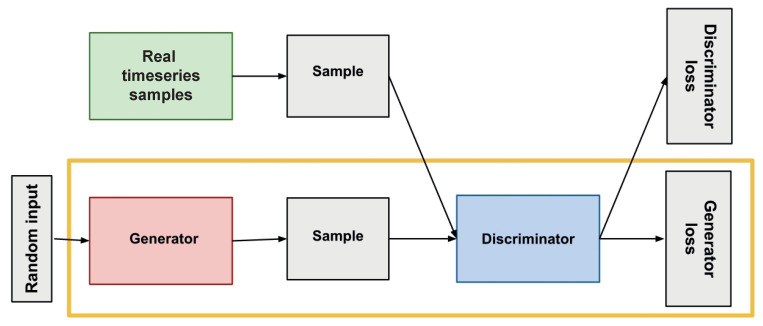
Overview of GAN structure.

Unsupervised anomaly detection using generative adversarial networks (GAN), as illustrated in Figure 7, involves training two models: a generator and a discriminator. The generator learns a model representing normal time series variability, while the discriminator learns to identify data points that do not conform to the normal behavior. The discriminator can then distinguish between time-series data corresponding to normal behavior. Moreover, using GANs for unsupervised anomaly detection in time-series data helps identify more complex anomalies than traditional methods since it can learn more complex dependencies between different variables within the time series (backpropagation).

### 4.2. Autoencoder (AE)

**Figure 8:**
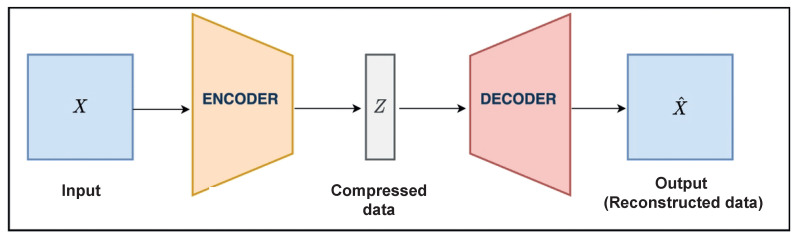
Overview of autoencoder network architecture.

Autoencoders are popular-deep learning-based anomaly detection models that have been observed to be reliable and effective. What makes this model so successful is its ability to reduce the time-series data to a lower dimension and reconstruct them in a higher dimension. As illustrated in Figure 8, this encoding and decoding can be performed with the help of different deep learning layers, such as convolutional layers. Then, the data are decoded to a higher dimension using an autoencoder algorithm. These reconstructed data can then be compared to the primary data to detect anomalies. Autoencoders have been a successful tool for anomaly detection in time-series data. Their efficiency can even be increased with the use of regularization, dropout, and noise-reduction techniques. Moreover, the autoencoder model is able to independently extract features from the input data, leading to better accuracy and lower computing costs.

### 4.3. Problem Formulation

Considering a time-series dataset $D=\left[X_{1},\;X_{2},\ldots ,\;X_{n}\right]\in R^{t\times n}$, where each $X_{i}=\left[x_{1i},\;x_{2i},\ldots ,\;x_{ti}\right]\in R^{t}$ (for i=1,…,n) comprising data obtained from sensors over a specific period of time t, the suggested model is trained with a subset of the dataset $D_{train}\in R^{t\times k}$ which only contains normal samples, where k is the number of training samples. The samples for testing are $D_{test}=\left[D_{test}^{v},D_{test}^{u}\right]\in R^{t\times \left(v+u\right)}$, with v and u representing the number of normal and abnormal samples, respectively, and n=k+v+u. Due to the imbalanced nature of the time-series data, the size of normal samples is greater than that of abnormal samples. A GAN-based model is trained with $D_{train}$ to reduce the output for each output $X_{i}$ in $D_{train}$. After the training process as shown in Appendix A, $D_{test}$ is passed through the trained model to evaluate it according to unseen samples during the learning. The trained generator can then generate fake normal samples from the noise vector. The discriminator model is used to accurately classify samples into normal or abnormal sequences. Autoencoders can be used as techniques for unsupervised or semi-supervised anomaly detection. To train an autoencoder, this technique involves training a model on a dataset that contains only normal data. We use a supervised learning approach by dividing the dataset into training and testing sets. The training Dtrain and testing Dtest sets are divided into input and output components by applying the sliding window approach. This approach uses the previous time intervals as both the input and output variables. This technique allows us to apply supervised learning to the autoencoder network. To evaluate the performance of the trained model, we used the reconstruction error, $L\left(x,\hat{x}\right)$, which measures the differences between the original input and the consequent reconstruction. The amount of discrepancy between a reconstructed representation and the original instance is used to calculate the anomaly score. The larger the difference, the greater the probability that it is an outlier. Moreover, the cutoff point for identifying anomalies can be adjusted by employing cross-validation to determine the most suitable threshold (Th) for the reconstruction error.

### 4.4. Dataset Preprocessing

The data used in this analysis were obtained from distinct sources, as shown in Table 3. These datasets were selected as they encompass soil and weather information and have the same hourly sampling rate to address the univariate anomaly detection and forecasting queries. The transformation for anomaly detection models involves three steps. Firstly, the time series are scaled between 0 and 1 using MinMaxScaler. This is an important step as it helps the network learn faster and converge with ease. Secondly, the time series is split into training, testing, and validation data. The training set consists of the first 80%, the testing set consists of the next 10%, and the validation set consists of the last 10%. Lastly, the training and testing splits are divided into input and output components by using the sliding window technique with sequence length 6 for anomaly detection models.

### 4.5. Implementation

We implemented a Generator network to produce fake samples. As shown in Table 3, it consists of one-dimensional convolution, LSTM, and dense layers with a binary cross-entropy loss function. Binary cross-entropy measures the performance of a binary classification model whose output is a probability value between 0 and 1. We implemented a Discriminator Neural Network that outputs a score for each sample, indicating whether it belongs to the real or fake sample. This Discriminator consists of one-dimensional convolution, LSTM, and dense layers. To obtain a probability value, a Sigmoid activation function is applied to the final layer. We implemented an Autoencoder Neural Network that tries to output the same inputs with less reconstruction error. This neural network consists of one-dimensional convolution, LSTM, and dense layers, followed by a dropout layer to help prevent overfitting. Adam optimization is a stochastic gradient descent method based on the adaptive estimation of first-order and second-order moments. In our case, we used the Adam optimizer implemented in the Keras/TensorFlow library, with a learning rate of 0.001 and β2 set to 0.5. GAN models are trained for 2000 epochs with a batch size of 128, and the autoencoder models are trained for 50 epochs with a batch size of 8. After each epoch, the model is stored on the hard drive to select the best model before overfitting occurs. The detailed network architecture of the autoencoder model can be found in Table 4, and the detailed network architecture of the GAN is presented in Table 5.

### 4.6. Description of Evaluation Metrics

After the learning process, the best model was selected and evaluated using balanced class validation data that contain 1000 normal and 1000 abnormal samples labeled by agronomy experts. Table 6 summarizes the evaluation metrics of the anomaly detection models related to soil moisture, air temperature, and air humidity.

The metrics used for evaluating the anomaly detection models include precision, recall, F1 score, and accuracy. They are calculated as follows:

$$\mathrm{Accuracy}=\frac{T_{p}+T_{n}}{T_{p}+f_{n}+f_{p}+T_{n}}\;\mathrm{Precision}=\frac{T_{p}}{T_{p}+f_{p}}\;\mathrm{Recall}=\frac{T_{p}}{T_{p}+f_{n}}\;F1-\mathrm{score}=2\times \frac{Precision\times Recall}{Precision+Recall}$$

- True Positive (TP): represents the number of abnormal samples correctly classified as attacks (correct detection).
- False Positive (FP): represents the number of normal samples wrongly classified as attacks (incorrect detection).
- True Negative (TN): represents the number of normal samples wrongly classified (correct detection).
- False Negative (FN): represents the number of abnormal samples wrongly classified as attacks (incorrect detection).
- Accuracy: reports the proportion of properly categorized samples to all other samples in the testing set.
- Precision: reports the percentage of samples properly categorized for all TP and FP in the testing set.
- Recall: the ratio of TP samples to the total number of TP and FN samples is known as recall.
- The F1-score reports the harmonic mean between precision and recall.

### 4.7. Results and Evaluation

**Figure 9:**
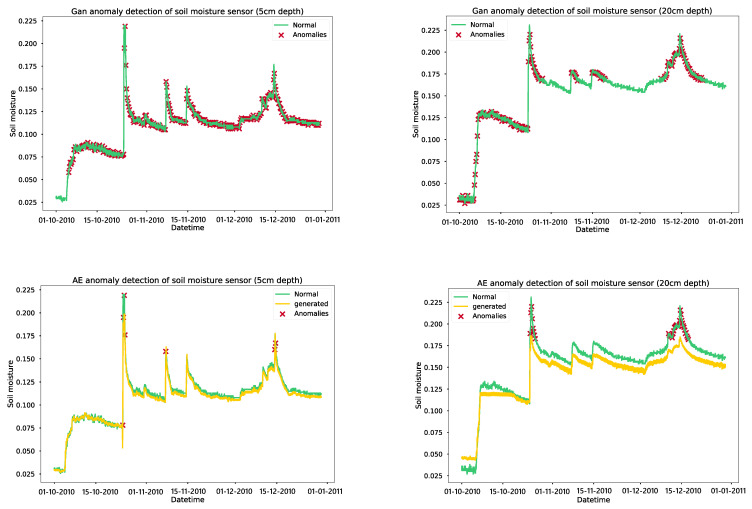
Results of anomaly detection models on the soil moisture parameter.

We trained a GAN model on soil moisture data for 2000 epochs, with an early stop mechanism implemented to stop training if the discriminator and generator losses remained low for 20 epochs. The GAN model for soil moisture at 5 cm depth converged at 500 epochs, while the model for 20 cm depth converged at 2000 epochs. Autoencoders were also trained for 100 epochs, with models saved to the hard drive after each epoch. The model before the overfitting occurred was selected, with the models at epoch 38 and epoch 20 being chosen for soil moisture models at depths of 5 cm and 20 cm, respectively. According to Table 6, the accuracy of each model ranged from 57.10% to 97.60%, while the F1 score ranged from 43.40% to 97.60%. Among the models, AE obtained the maximum accuracy and F1 score of 97.60% and 97.60%, respectively, while GAN achieved accuracy and F1 scores of 87.50% and 88.88%, respectively. The autoencoder error thresholds were set according to the 95% percentile of training data. As shown in Figure 9, we chose four-month time-series data from a soil moisture dataset, covering the time period from October 2010 to January 2011.

**Figure 10:**
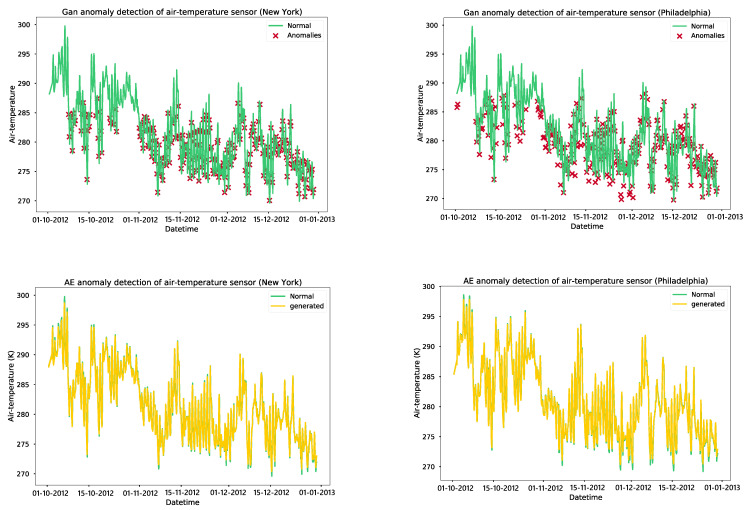
Results of anomaly detection models on the air temperature parameter.

The GAN anomaly detection model identifies many anomalies in the normal behavior of soil moisture sensors, which may lead to false alarms for the farmer. On the other hand, the autoencoder model only picks up anomalies in the curves’ peaks that result from irrigation or rainfall. We trained a GAN model for detecting anomalies in air temperature for 3000 epochs and added an early stopping feature that would stop the training if the loss for the generator and discriminator remained low for 20 consecutive epochs. The training of the GAN model for air temperature in New York was observed to converge at 2700 epochs, while the training of the model for Philadelphia converged at 1870 epochs. Additionally, we also trained autoencoders for 100 epochs, saving the models after every epoch. The model that was trained before overfitting occurred was selected, with the models at epochs 95 and 87 chosen for air temperature models in New York and Philadelphia, respectively. The error thresholds for the autoencoder models were set based on the 95th percentile of the training data. This resulted in high accuracy, recall, and F1 scores for the autoencoder models compared to the GAN models, with the precision being similar for both. As illustrated in Figure 10, we selected four-month time-series data from an air temperature dataset, from October 2012 to January 2013. The GAN anomaly detection model detects abnormalities in the normal behaviour of the air temperature sensor, whereas the autoencoder model recognizes the typical pattern of the air temperature sensor and does not detect any anomalies.

**Figure 11:**
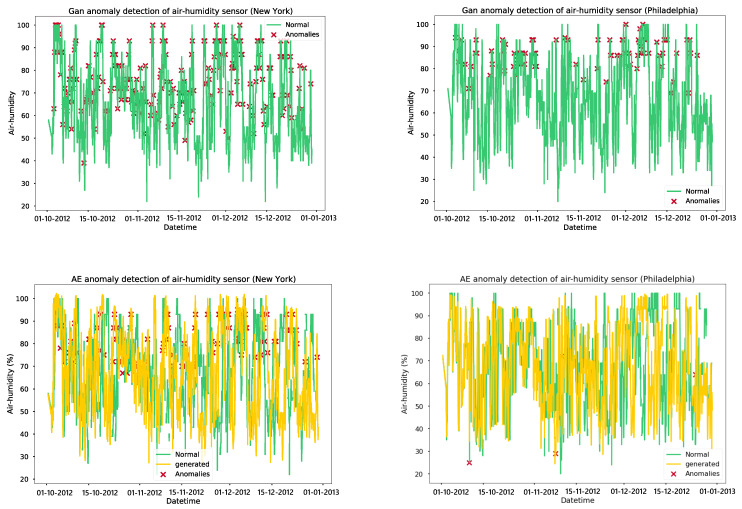
Results of anomaly detection models on the air humidity parameter.

**Figure 12:**
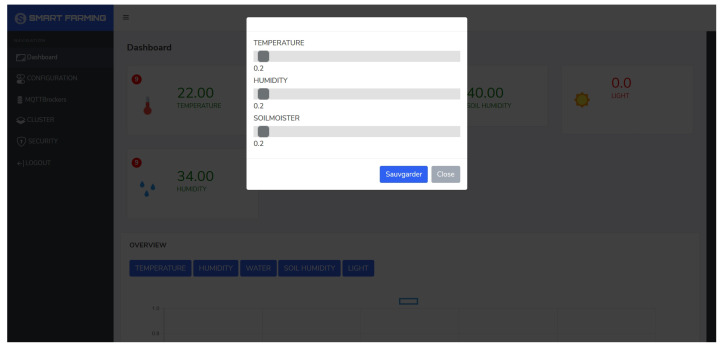
Configuring anomalies and error thresholds in the IoT gateway web application.

We trained a GAN model for anomaly detection in air humidity for 2000 epochs with an early stopping mechanism in place to stop training if the discriminator and generator losses remained low for 20 epochs. The GAN model for air-humidity in New York converged at 680 epochs, while the model for Philadelphia converged at 1412 epochs. Additionally, autoencoders were trained for 100 epochs, and the models were saved after each epoch. The model before overfitting occurred was chosen, with the models at epochs 93 and 100 selected for air humidity autoencoder models in New York and Philadelphia, respectively. This resulted in high accuracy, precision, recall, and F1 scores for the autoencoder models, which performed better than the GAN models. The data used in Figure 11 were a four-month time series of air humidity from October 2012 to January 2013. The GAN model identifies anomalies in the normal behavior of the sensor, while autoencoders recognize the normal patterns of the data and detect only a few anomalies. As per the model validation results, the autoencoder-based models demonstrated high accuracy and precision in detecting anomalies compared to the GAN models. To optimize the models without sacrificing accuracy, they were converted using TensorFlow Lite and deployed on IoT gateways. An API and a simple web UI were established to configure the anomaly error thresholds and alert end users of any sensor malfunctions, as presented in Figure 12.

## 5. Irrigation Factors Forecasting Using Deep Learning

**Figure 13:**
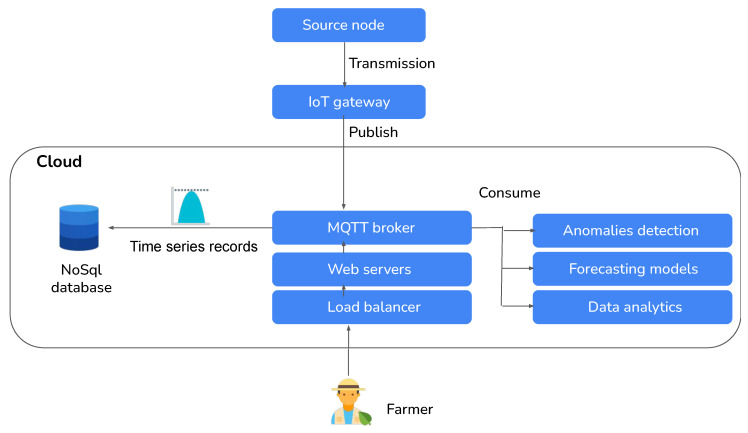
Illustration depicting the interaction between end users and cloud services within a system.

As illustrated in Figure 13, the developed system incorporates various microservices, including anomaly detection, forecasting, and data analytics, to enhance farmers’ ability to make irrigation decisions. These microservices are exposed through the system. To provide the farmer with the necessary tools for monitoring and forecasting climate and soil measurements, we have implemented a time-series NoSQL database to efficiently handle the large volume of sensor data. This type of database is particularly well suited for managing time-dependent data and enabling efficient manipulation operations. Additionally, it supports auto-scaling capabilities, ensuring that the system can handle increased data loads if required. To provide accessibility to the system, we have deployed multiple web servers that host web applications.

This allows the farmer to access the system and its functionalities through a user-friendly interface. To an ensure optimal performance and distribution of user requests, load balancers are implemented to evenly distribute the incoming traffic across the deployed web servers. In the next experiment, we will test the accuracy of the soil and weather forecasting models using autoencoders. In the first stage, we will feed the autoencoders’ anomaly detection models with data from the datasets and replace detected anomalies with the reconstructed inputs. The obtained data are structured into sequences in the next stage by applying the sliding window approach. This methodology improves those observed in previous studies [3,28] when integrated with an IoT platform, as a tool for simplifying the labeling of actual anomalies, and can assist in the creation of supervised datasets for future research in the field. Prediction models based on deep learning techniques can also correct anomalies. We use the previous 24 records as inputs and the next 24 as outputs. This technique allows us to apply supervised learning to soil and weather parameter forecasting tasks.

### 5.1. Forecasting Model for Potential Irrigation Factors

Based on the previous comparison of generative adversarial networks (GANs) and autoencoders (AEs) for finding anomalies, it was found that AEs are more accurate and have a lower rate of false alarms. As a result, AE models were chosen for the data collection process. This section aims to evaluate the performance of the forecasting models after applying data clearing and correction techniques, which are intended to enhance the data’s quality and reliability for accurate forecasts. Table 6 shows the architecture of the model. This architecture is a sequence-to-vector transformation, where the input sequence goes through a series of operations to pull out features and capture temporal relationships. This process involves using both convolutional and LSTM layers. The convolutional layers extract pertinent features from the input sequence, while the bidirectional LSTM architecture processes the input sequence in both forward and backward directions, enabling the model to capture past and future context simultaneously. Finally, the output of the LSTM layer is fed into a fully connected layer for additional processing and to generate the final output of the model. This architecture enables the model to effectively analyze and understand the input sequence, producing a condensed vector representation as the ultimate result.

### 5.2. Evaluation Metrics of Forecasting Models Based on Deep Learning Models

When analyzing prediction models of future measurement performance, the following metrics are typically used: Mean Absolute Error (MAE), Mean Square Error (MSE), and Root Mean Square Error (RMSE) are the three most commonly used loss metrics to evaluate the performance of DL models related to regression problems. They are given as follows:

$$\begin{array}{cc} \mathrm{MSE} & =\sum_{i=1}^{N}{\left(x_{i}-y_{i}\right)}^{2}\\ \mathrm{MAE} & =\sum_{i=1}^{N}\left|x_{i}-y_{i}\right|\\ \mathrm{RMSE} & =\sqrt{\sum_{i=1}^{N}{\left(x_{i}-y_{i}\right)}^{2}} \end{array}$$

### 5.3. Results and Discussion

According to our empirical study, the Adam optimizer from Keras/TensorFlow with a learning rate of 0.001 was used for training the CNN/BiLSTM models for 200 epochs using a batch size of 32. The model was saved after each epoch to pick the best model before overfitting occurred. Table 7 displays the in-depth structure of the CNN/BiLSTM models. The model inputs a sequence of 24 previous values to predict the next 24 subsequent sensor measures. As shown in Table 8, the evaluation results for the soil moisture forecasting model are presented for two different depth sensors (sm_5 and sm_20). For sm_5, when no anomalies were replaced, the model achieved an MSE of 1.2273, an RMSE of 1.1078, and an MAE of 0.999. However, a significant performance improvement was observed when 1025 anomalies were replaced, resulting in a notable decrease in MSE to 0.272243, RMSE to 0.52176, and MAE to 0.4703. These findings demonstrate that replacing anomalies positively impacted the model’s ability to forecast soil moisture at sm_5 sensors, leading to more accurate predictions. In the case of sm_20, when no anomalies were replaced, the model yielded an MSE of 1.70128, an RMSE of 1.3043, and an MAE of 1.234.

**Figure 14:**
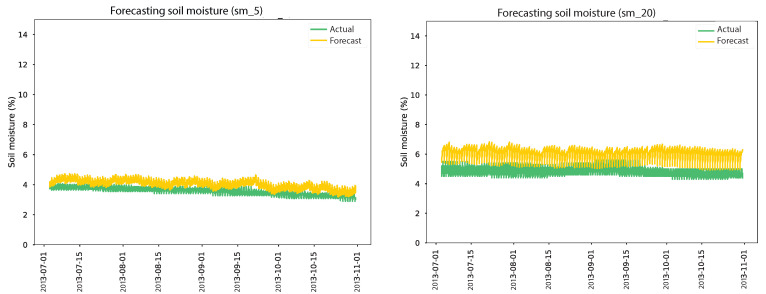
Results of soil moisture forecasting models over four months.

Subsequently, when 716 anomalies were replaced, a slight performance improvement was observed, resulting in a reduced MSE of 0.8740, an RMSE of 0.9348, and an MAE of 0.861. The decrease in the MSE, RMSE, and MAE values indicates enhanced accuracy in the model’s predictions for sm_20 sensors, although the improvements were comparatively smaller than those observed for sm_5. These results underscore the significance of replacing anomalies in the dataset to improve the forecasting accuracy of the model. The improvements achieved for the sm_5 sensors demonstrate the substantial impact of addressing anomalies on the model’s performance. However, for the sm_20 sensors, the improvements were relatively modest, suggesting that anomalies had a lesser effect on the model’s predictions in this particular case. According to the results, the autoencoder model trained on clean data demonstrated the best forecasting performance, as shown in Figure 14. The actual and expected soil moisture were nearly identical, with minor discrepancies at the 5 cm and 20 cm depths. Table 9 presents the evaluation of an air temperature forecasting model for New York and Philadelphia, considering scenarios with and without anomaly replacement. The air temperature model in New York, not replacing anomalies, resulted in an MSE of 20.1318, an RMSE of 4.4868, and an MAE of 3.497. However, when 1140 anomalies were replaced, the model’s performance improved significantly, with the MSE decreasing to 17.7633, the RMSE decreasing to 4.2146, and the MAE decreasing to 3.436. This suggests that replacing anomalies enhanced the model’s accuracy in predicting air temperature in New York.

**Figure 15:**
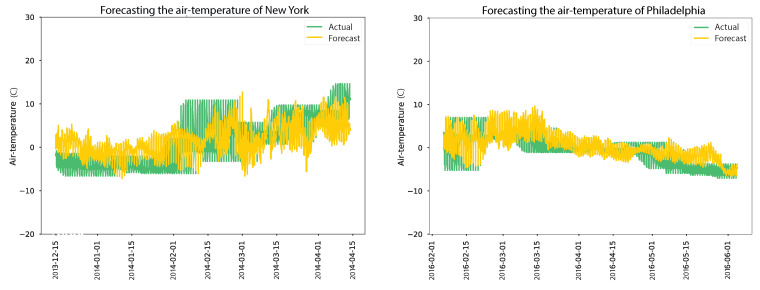
Results of air-temperature forecasting models over four months.

Similarly, for air temperature in Philadelphia, the model achieved an MSE of 16.2342, an RMSE of 4.0291, and an MAE of 3.3 when anomalies were not replaced. After replacing anomalies, the model’s performance improved further, resulting in an MSE of 4.635611, an RMSE of 2.1530, and an MAE of 1.648. This indicates that anomaly replacement positively influenced the model’s forecasting accuracy in Philadelphia. Overall, the results underscore the significance of anomaly replacement in improving the accuracy of the air temperature forecasting model, as depicted in Figure 15. The findings demonstrate that addressing anomalies led to more precise predictions for air temperature in both New York and Philadelphia. As indicated in Table 10, the evaluation results of an air humidity forecasting model for two locations are as follows. In New York and Philadelphia, the model’s performance was assessed under different scenarios where anomalies were either not corrected (corrected anomalies = 0) or replaced using a correction model. In the case of air humidity forecasting in New York, the results show that replacing anomalies had a minor positive effect on the model’s performance. Although the improvements in MSE, RMSE, and MAE values were relatively small, they indicate a slight increase in the accuracy of the model’s predictions after anomaly replacement.

**Figure 16:**
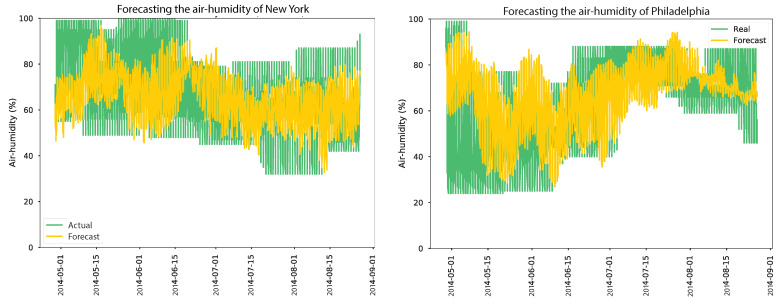
Results of air-humidity forecasting models over four months.

**Figure 17:**
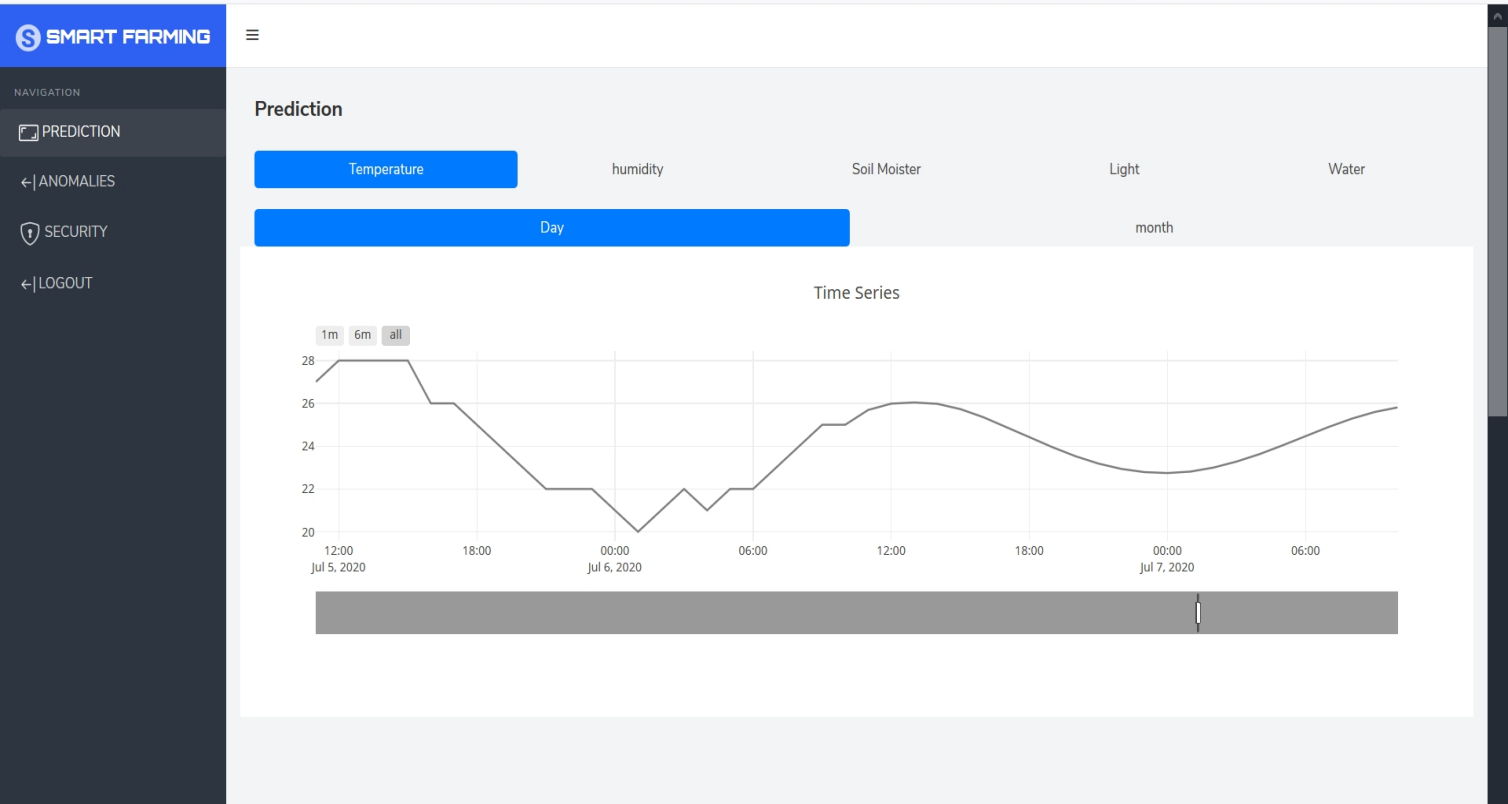
Estimating irrigation measures using a web application accessible online.

Similarly, for air humidity forecasting in Philadelphia, replacing anomalies had a limited impact on the model’s performance. The reduction in MSE, RMSE, and MAE values was minimal, suggesting only a slight improvement in the accuracy of the model’s predictions. Overall, the results indicate that replacing anomalies had a limited influence on the performance of the air humidity forecasting model in both New York and Philadelphia, as shown in Figure 16. Anomalies may have a relatively small effect on the accuracy of the model’s predictions for air humidity in these locations. However, the long-term forecast error was significantly high due to various factors that can impact air humidity patterns, such as air temperature, air pressure, wind speed, and air pollution. After validating the models obtained, they were converted using TensorFlow Lite to improve efficiency without sacrificing accuracy and deployed on the cloud. An API and web dashboard were developed to display and predict weather and soil parameters, as shown in Figure 17.

## 6. Conclusions and Future Trends

This article introduces version 1.0 of the INTEL-IRRIS low-cost and fog-IoT/AI system, developed by the University of Oran 1 for SFCs. We present an IoT-based smart farming system that integrates anomaly detection based on autoencoders, demonstrating superior performance compared to GANs with accuracy rates of 90% for soil moisture, 95% for air temperature, and 97% for air humidity. Additionally, anomalies can be adjusted using reconstructed inputs from autoencoder models. The results showcase minor estimation errors for soil moisture and air temperature hourly forecasting models, indicating the effectiveness of our approach. The proposed solution improves the effects observed in previous studies and can also assist in creating supervised datasets for future research in the field. This platform will need more research that takes into consideration several perspectives:

1. To enhance the accuracy of the acquired data, we will increase the accuracy of low-cost sensors using autonomous and remotely managed techniques to improve the different sensors’ calibration [32]. As a consequence, our water–soil–plant–climate interaction models will prescribe more correct measures.
2. To enhance the reliability, accessibility, and tracking of sensors and actuators, incorporating a blockchain security framework is recommended. This integration will establish a strong mechanism for data integrity, availability, and traceability in the system.
3. The design and implementation of precision PA-based Low-Power Wide-Area Network (LPWAN) technologies like Lora and Sigfox. These approaches are designed to conserve energy, like the Long-Range Wide-Area Network (LoRaWAN), and enhance the irrigation efficiency of large-holder farmers.
4. Offer the “out-of-the-box” function, meaning that the control component that recommends irrigation is integrated into the IoT gateway and, as a result, does not need an internet connection, as implemented by WAZIUP partner [27].
5. Traditional deep learning systems need centralized data gathering and processing, which is becoming more unfeasible due to efficiency issues and rising data privacy concerns. Due to these qualities, federated learning has been a hot topic in smart agriculture. We intend to conduct a comprehensive assessment of the FL approach and the centralized machine learning models.

## Figures and Tables

**Table 2:** The proposed platform cost.

Equipements	Components	Price
Gateway	Raspberry Pi 3 B+ + SD card 16 GB	USD 125
IoT device	Arduino Mega card + Arduino UNO REV3 LCD display nokia + LCD display I2C LEDs + jumpers	USD 63
Antenna and related components	We use NRF module with antenna Solar panel ZW85X115-12 + solar panel 6 V amplifier + power supply 9 V + Power supply 12 V	USD 31
Physical sensors	DHT22 + moisture sensor V2.0 Water level sensor + light sensor	USD 17
Actuators	Water pump + relay switch Water flow sensor	USD 24
	Total	USD 260

**Table 3:** Description of datasets used to train deep learning models.

Datasets	Parameters	Columns	Unity	Scale	Missing Data	Traning Data	Testing Data
[30]	Soil Moisture	sm_5	%	from 6 to 39.7	719	27,216	3024
		sm_20	%	from 2.79 to 30.1	719	27,216	3024
[31]	Air-temperature	New York	kelvin	from 250.77 to 310.24	793	14,287	1587
		Philadelphia	kelvin	from 250.39 to 308.0	3	31,104	3456
[31]	Air-humidity	New York	%	from 10 to 100	1624	15,552	1728
		Philadelphia	%	from 10 to 100	624	15,552	1728

**Table 4:** Network architecture of AE model.

Layer	Output Size
Input	6
Conv1D	3 × 32
Dropout	3 × 32
Conv1D	2 × 16
Conv1DTranspose	4 × 16
Dropout	4 × 16
Conv1DTranspose	8 × 32
Conv1DTranspose	8 × 1
Flatten	8
FC	6

**Table 5:** Network architecture of GAN model.

Layer	Output Size
**Discriminator**	
Input	6
Conv1D	5 × 32
LeakyReLU	5 × 32
Conv1D	3 × 64
Batch Normalization	3 × 64
LeakyReLU	3 × 64
LSTM	16
FC (Sigmoid)	1
**Generator**	
Input	6
Conv1D	5 × 32
LeakyReLU	5 × 32
LSTM	128
FC	6

**Table 6:** Evaluation metrics of anomaly detection models.

Parameters	Columns	AE Evaluation Metrics				GAN Evaluation Metrics			
		Accuracy	Precision	Recall	F1	Accuracy	Precision	Recall	F1
Soil moisture	sm_5	0.93	0.93	0.94	0.93	0.875	0.875	1.0	0.8888
	sm_20	0.935	0.9306	0.94	0.935	0.635	0.965	0.28	0.434
Air temperature	New York	0.932	0.8802	1.0	0.936	0.75	0.93	0.54	0.6835
	Philadelphia	0.9595	0.9563	0.963	0.959	0.6785	0.92	0.391	0.548
Air humidity	New York	0.9735	0.949	1.0	0.974	0.6905	0.7671	0.547	0.638
	Philadelphia	0.976	0.954	1.0	0.976	0.571	0.543	0.895	0.6759

**Table 7:** Network architecture of parameter forecasting model.

Layer	Output Size
Input	24
Conv1D	12 × 32
MaxPooling1D	6 × 32
Bidirectional LSTM	256
FC	24

**Table 8:** Evaluation of soil moisture forecasting model.

Parameter	Columns	Corrected Anomalies	Evaluation Metrics		
			MSE	RMSE	MAE
Soil moisture	sm_5	0	1.2273	1.1078	0.999
	sm_5	1025	0.272243	0.52176	0.4703
	sm_20	0	1.70128	1.3043	1.234
	sm_20	716	0.8740	0.9348	0.861

**Table 9:** Evaluation of air temperature forecasting model.

Parameter	Columns	Corrected Anomalies	Evaluation Metrics		
			MSE	RMSE	MAE
Air temperature	New York	0	20.1318	4.4868	3.497
	New York	1140	17.7633	4.2146	3.436
	Philadelphia	0	16.2342	4.0291	3.3
	Philadelphia	519	4.635611	2.1530	1.648

**Table 10:** Evaluation of air humidity forecasting model.

Parameter	Columns	Corrected Anomalies	Evaluation Metrics		
			MSE	RMSE	MAE
Air humidity	New York	0	200.26	14.15	10.73
	New York	145	186.104	13.642	10.57
	Philadelphia	0	219.91	14.829	11.53
	Philadelphia	86	217.485	14.747	11.35

**Figure A1:**
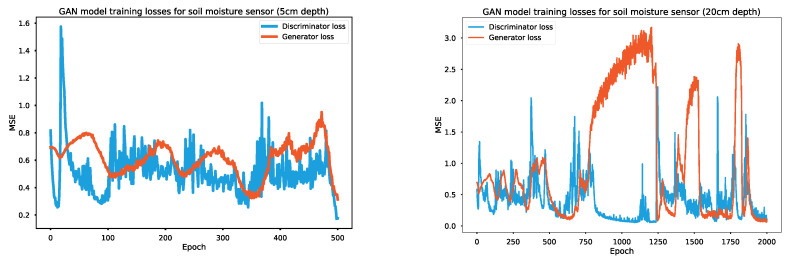

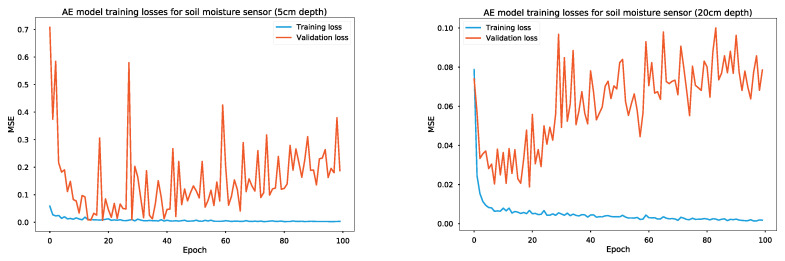
Training evolution of soil moisture anomaly detection models.

**Figure A2:**
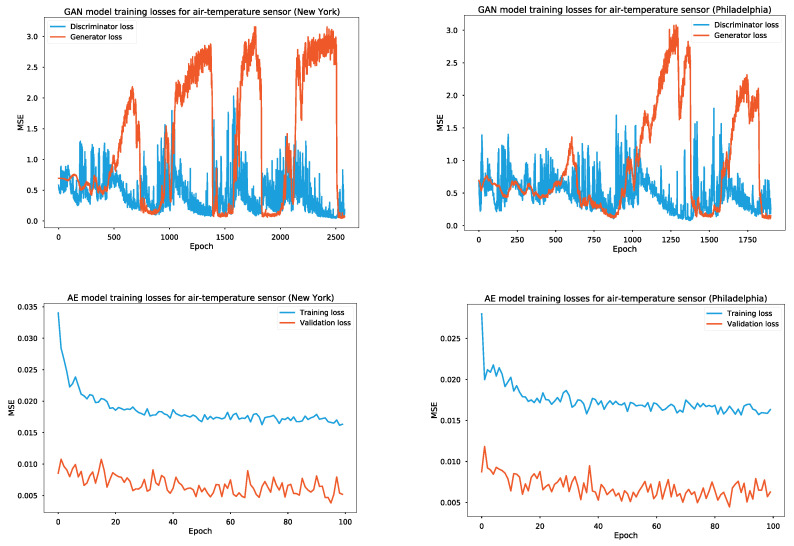
Training evolution of air temperature anomaly detection models.

**Figure A3:**
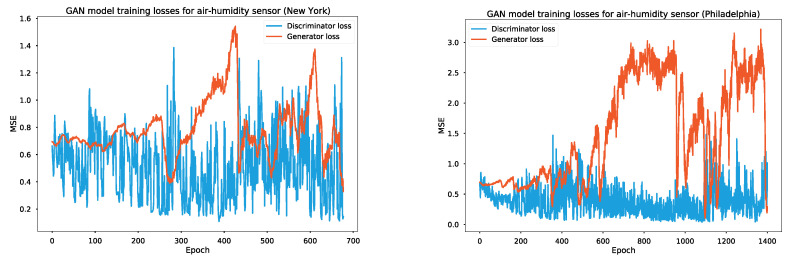

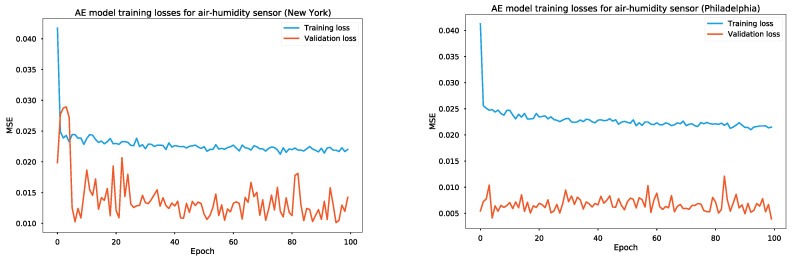
Training evolution of air humidity anomaly detection models.

## Data Availability

The datasets used and/or analyzed during the current study are available from the corresponding authors upon reasonable request.

## Abbreviations

The following abbreviations are used in this manuscript:

IoT	Internet of Things.
DL	Deep Learning.
ML	Machine Learning.
AE	Autoencoder.
GAN	Generative Adversarial Network.
ANN	Artificial Neural Network.
RNN	Recurrent Neural Network.
LSTM	Long Short-Term Memory.
BiLSTM	Bidirectional Long Short-Term Memory
CNN	Convolutional Neural Network.
SFCs	Smallholder Farmer Communities.
